# Comparison of Free Flavonoids and the Polyphenol Content in the Bran of a Newly Developed Sorghum Variety and Two Commercially Available Sorghum Varieties

**DOI:** 10.3390/metabo14110628

**Published:** 2024-11-15

**Authors:** Mariely Cristine Dos Santos, Naoki Tanaka, Shigemitsu Kasuga, Kazuhiro Tanabe, Chihiro Hayashi, Masahiro Mizuno, Yoshihiko Amano

**Affiliations:** 1 Department of Biomedical Engineering, Graduate School of Medicine, Science and Technology, Shinshu University, 4-7-1 Wakasato, Nagano City 380-8553, Nagano Prefecture, Japan; 22hb103a@shinshu-u.ac.jp (M.C.D.S.); m-mizuno@shinshu-u.ac.jp (M.M.); 2 Department of Global Medical Research Promotion, Graduate School of Medicine, Shinshu University, 3-1-1 Asahi, Matsumoto City 390-8621, Nagano Prefecture, Japan; naopi@shinshu-u.ac.jp; 3 Institute of Agriculture, Academic Assembly, Shinshu University, 8304 Minamiminowa, Matsumoto City 99-4598, Nagano Prefecture, Japan; skasuga@shinshu-u.ac.jp; 4 Medical Solution Promotion Department, Medical Solution Segment, LSI Medience Corporation, Tokyo City 174-8555, Tokyo Prefecture, Japan; kazuhirotanabe77@gmail.com (K.T.); hayashi.chihiro@ma.medience.co.jp (C.H.); 5 Institute of Engineering, Academic Assembly, Shinshu University, 4-17-1 Wakasato, Nagano City 380-8553, Nagano Prefecture, Japan

**Keywords:** sorghum bicolor, polyphenol, flavonoids, untargeted metabolomics, newly developed variety

## Abstract

**Background/Objectives**: *Sorghum bicolor* is a source of many bioactive components, such as polyphenols. Those components are present mainly in its bran, often removed in industrial processes through decortication. In that sense, this work aimed to analyze the polyphenol content, especially free flavonoids, from the bran of a newly developed variety compared to other commercially available varieties. **Methods:** The samples were white sorghum TDN^®^ Sorgho, red sorghum Mini Sorgho, and the newly developed red sorghum RILN-156. First, decortication was conducted to obtain the bran samples, which were triturated and then sieved. The use of colorimetric methods allowed the general quantification of the polyphenolic components. First, the polyphenol content was extracted using 70% methanol. Then, the samples’ total phenolic content, total flavonoid content, total tannin content, total anthocyanin content, and antioxidant potential were determined. To analyze the different components and identify the free flavonoids, an untargeted metabolomics analysis (with liquid chromatography coupled with mass spectrometer (LC/MS) and capillary electrophoresis coupled with a mass spectrometer (CE/MS)) was performed. **Results:** The results have shown that apart from anthocyanin and tannin, the newly developed variety, RILN-156, presented the highest concentration of polyphenolic content, including a higher antioxidant capacity. The exploratory analysis identified 19 flavonoids within the samples, with galangin and daidzein being the most abundant ones. **Conclusions:** These results show a promising finding for using this newly developed sorghum variety (RILN-156) industrially and further investigating its health benefits. They also elucidate the differences between colored sorghum within themselves and with white sorghum varieties.

## 1. Introduction

Sorghum (*Sorghum bicolor* L.) is a drought-tolerant, nutritious cereal crop highly produced worldwide. As the fifth most-produced cereal globally, it is popular in the bioenergy industry and as a feedstock material [1]. According to their purpose, there are four main types of sorghum: sweet sorghum, grain sorghum, forage sorghum, and bioenergy sorghum [2]. Besides these versatile applications, sorghum is also beneficial for cultivation, as it efficiently uses water and converts solar energy [3]. Additionally, its extensive root system assists the preservation of biodiversity in its growing environment [1].

Dietary-wise, grain sorghum presents many interesting nutritional traits, standing out among other cereals. The grain is a source of carbohydrates, non-starch polysaccharides, fat, proteins, bioactive components, and micro and macronutrients. Furthermore, providing an amount of dietary fiber that could supply the recommended daily intake and being gluten-free, this grain appeals to the most variable consumer groups [4].

The grain can be divided into pericarp, sometimes accompanied by a pigmented testa, endosperm, and germ. Each of these fractions is the source of different nutrients. The germ contains a substantial amount of lipids and vitamins, especially from the B-complex, which is fat-soluble, and minerals. As for the endosperm, besides being rich in starch, it also has compounds like proteins, vitamins from B-complex, and minerals. Lastly, the pericarp and testa are a great source of bioactive compounds such as non-starch polysaccharides, phenolic acid, carotenoids, and phenolic compounds [5].

Phenolic compounds, such as flavonoids, are relevant secondary metabolites in plants that play important roles, such as defense against biotic and abiotic stress, coloring, regulation of auxin transport, etc. Flavonoids are divided according to the differences in their chemical structure and can be classified into flavonols, flavones, isoflavones, anthocyanidins, flavanones, flavanols, and chalcones. They have been reported to present many health benefits due to their antimicrobial, antioxidant, anticancer, and anti-inflammatory properties [6].

Although the sorghum grain is rich in bioactive components, it is still overseen as a food product in many Western countries, where it is used as animal feed. This is primarily because of the unwanted sensorial characteristics some varieties might have. An example is the bitter taste of dark-colored sorghum varieties [7]. In this case, the bitter taste is caused by phenolic components, such as tannins, present in the pericarp of this grain, like most of the phenolic components from cereals [8,9]. Even in countries where sorghum is used in the human diet, the pericarp is one of the grain’s discarded parts during decortication processing. The pericarp, the aleurone layer, and the seed coat form the sorghum bran [10].

In the last decades, studies have investigated the benefits of sorghum bran, previously considered industrial waste, to make the most of the essential bioactive components in this matrix. Among the beneficial components of sorghum bran, the previously mentioned phenolic compounds are gaining crescent attention [11,12,13,14]. Sorghum bran has colored varieties, and each color influences the profile of the grain’s phenolic components [15]. The class of flavonoids is one of the protagonists for the colors of plants [16].

With the challenges in unraveling the structure of sorghum and its bioactive components, researchers and farmers are in search of ideal hybrids for different environments and applications, which will expand the variations in phenotypic and structural characteristics among the different sorghum [17,18,19,20,21,22].

Nagano Prefecture, a region in central Honshu, Japan, has been investing in cultivating and promoting sorghum as a food ingredient. Some projects not only encourage the development of new recipes but also new forms of utilization of the grain and its byproducts and the development of new varieties. Exploring new applications of this matrix, aiming to avoid the waste of resources rich in added-value compounds, makes further investigation necessary on which beneficial compounds can be found within the grain.

In this sense, this research aims to compare the differences in the polyphenolic content, focusing primarily on the identification of the flavonoid profile of sorghum bran from two different varieties (TDN^®^ Sorgho and Mini Sorgho) and a newly developed variety (RILN-156).

## 2. Materials and Methods

### 2.1. Sorghum Samples

Three varieties of sorghum were used for this study. Two of them are abundantly cultivated in Nagano Prefecture and are commercially available seeds: white sorghum TDN^®^ Sorgho and red sorghum Mini Sorgho sold by KANEKO SEEDS Co., Ltd., (Maebashi, Japan). The third variety used was red sorghum RILN-156, produced by Shinshu University, Ina City, Nagano Prefecture, Japan.

This recombinant inbred line (RIL) was developed in 2005, aiming to genetically analyze grain characteristics and obtain a high-quality grain-type edible for human consumption. The cultivation was conducted with the Takakibi x M36001 strain. Then, the F1 generation was trained in 2005, expanded as a population in 2006, and 227 strains were cultivated from 227 individuals.

Subsequently, random seeds without genetic manipulation were collected from F1 to continue the generation. In 2018, when generation F8 was reached, the breeding process as a recombinant inbred line was completed. RILN-156 and every other RIL obtained were evaluated according to their main agronomic traits and grain quality. Later, RILN-156 was provided for the current investigation of its polyphenol profile.

#### Preparation of Sorghum Bran

The seed was milled with a household rice milling machine (SM—500W, MK-SEIKO Co., Ltd., Chikuma, Japan) using the program according to the amount of sample, which takes around 1 min and 50 s to finish, to obtain the bran from each grain. These milling conditions were performed three times for each sample. After that, the bran was collected and sieved with a mesh (opening: 250 μm, diameter: 160 mm). The remaining larger bran fractions were triturated with a blender (Wonder Blender WB-1, Osaka Chemical, Osaka, Japan) and further sieved with the same mesh mentioned above. The bran was stored in a sealed container until further analysis.

After the samples were chosen, first they were pre-analyzed with chemical colorimetric methods to characterize and access the feasibility of analyzing them by LC/MS and CE/MS.

### 2.2. Reagents Specifications

Methanol ACS reagent, ≥99.8% from Merck^®^ (Darmstadt, Germany) Folin–Ciocalteu reagent, sodium carbonate, gallic acid from Merck^®^, aluminum chloride, potassium acetate, quercetin from Merck^®^, potassium chloride, sodium acetate, hydrochloric acid, luteolin and apigenin from Merck^®^, butanol, iron (III) ammonium sulfate, catechin from Merck^®^, 2,2-Di(4-tert-octylphenyl)-1-picrylhydrazyl, free radical, and Trolox from Merck^®^.

### 2.3. Extraction of Polyphenol from Sorghum Bran

For the extraction, 70% methanol was used, as described by Zhang et al. [23]. The procedure was carried out according to the protocol first described by Awika et al. [24] and later adapted by Tyagi et al. [25]: 2.5 g of sorghum bran were added to 20 mL of the solvent (methanol 70%) and shaken for 2 h at 25 °C in a 50 mL conical centrifuge tube. The mixture was then left at a −20 °C freezer overnight for the dispersion of phenolics to occur. Subsequently, the samples were centrifuged at 7000× *g* for 10 min, and the supernatant was collected. The precipitate was washed twice (10 mL) with methanol 70%, and the supernatant was collected, mixed, and stored at −20 °C until further analysis. As the colorimetric analyses were conducted without solvent evaporation, the final concentration was corrected according to the dilution of the sample in methanol for the extraction.

#### 2.3.1. Determination of Total Phenolic Content

The total phenolic content (TPC) was determined following the Folin–Ciocalteu method [26]. The assay was conducted on a microplate adapted from Zhang et al. [27]. An amount of 20 μL of the extract dilutions were mixed into 100 μL of Folin–Ciocalteu 3% solution. After waiting 5 min, 80 μL of sodium carbonate 2% was added, and the solution was incubated for 30 min at room temperature in the darkness. The absorbance was read at 760 nm, and since gallic acid was used for the calibration curve, the results were expressed in mg of gallic acid equivalent (GAE mg/100g).

#### 2.3.2. Determination of Total Flavonoid Content

The total flavonoid content (TFC) was determined using Chang et al.’s aluminum-chloride colorimetric method [28], adapted to microplates. The original method involves mixing 0.1 mL of samples with 1.5 mL of 95% ethanol, 0.1 mL of 10% aluminum chloride, 0.1 mL of 1 M potassium acetate, and 2.8 mL of deionized water. The mixture was incubated for 40 min at room temperature, and the absorbance was read at 415 nm. The results were expressed in mg of quercetin equivalent (QE mg/100 g).

#### 2.3.3. Determination of Total Anthocyanin Content

The total anthocyanin content (TAC) was determined using the pH differential method [29]. Buffers with pH 1 (125 mL of 0.2 N potassium chloride, 385 mL of 0.2 N hydrochloric acid, 490 mL of distilled water) and pH 4.5 (400 mL of 1 M sodium acetate, 240 mL of 1 N hydrochloric acid and 360 mL of distilled water) were made. Then, 200 μL of the sample was mixed with 800 μL of the respective buffer and incubated in the darkness for 2 h at room temperature. First, the molar absorption coefficient of both apigenin and luteolin was calculated using the Beer–Lambert Law:

(1) A=ε×c×l

*A* = absorbance; *ε* = molar absorption coefficient (M^−1^ × cm^−1^); *c* = molar concentration (M); *l* = optical path length (cm).

After discovering these values (27.38 for apigenin and 19.98 for luteolin), the Beer–Lambert Law could be rearranged to find the total anthocyanin content based on the concentration of luteolin and apigenin in the samples.

(2) $$\gamma =\frac{A}{\varepsilon \times l\times 10^{3}\times M\times DF}$$

*A* = absorbance at 270 nm (pH 1−pH 5); *ε* = molar absorption coefficient (M^−1^ × cm^−1^); *l* = optical path length (1 cm); *M* = molecular mass of anthocyanin standard; *DF* = dilution factor.

Absorbance was read at 270 nm. Luteolin and apigenin were used as the standards [21].

#### 2.3.4. Determination of Total Tannin Content

Total condensed tannin (TCT) was determined by the butanol-hydrochloric acid method [30]. In this method, 0.5 mL of sample is added to 3 mL of butanol-hydrochloric acid and 0.1 mL of iron (III) reagent solution (iron (III) ammonium sulfate in 2 M hydrochloric acid). The reaction solution is incubated at 100 °C for 60 min, and the absorbance is read at 450 nm. The standard curve was conducted with catechin; the results are expressed in mg of catechin equivalents (CE) per gram of sample.

#### 2.3.5. DPPH Scavenging Assay

The scavenging activity was determined by the DPPH (2,2-diphenyl-1-picrylhydrazyl) assay, following Enkhtsetseg et al. [31] adaptation on Adedapo et al. [32]. An amount of 100 μL of the sample was mixed with 2.9 mL of 0.1 mM DPPH–methanol solution and incubated in the darkness for 30 min. The absorbance was read at 517 nm, using methanol as a reference and methanol 70% as the control sample. Trolox was used for the calibration curve, so results were expressed in μmol trolox equivalents (TE) per g of sample.

### 2.4. Untargeted Metabolomic Analysis of Sorghum Bran

#### 2.4.1. Samples for Liquid Chromatography Coupled with Mass Spectrometer (LC/MS) and Capillary Electrophoresis Coupled with Mass Spectrometer (CE/MS)

For the untargeted metabolomics analysis, samples grown in different cities of Nagano Prefecture and districts of Nagano City were collected: TDN^®^ Sorgho from three locations (Iizuna Town, Naniai, Nagano City and Wakaho, Nagano City), Mini Sorgho from four locations (Chikuma City, Shiojiri City, Suzaka City, and Nagano City), and RILN-156 from four locations (Suzaka City, Ueda City, Naniai, Nagano City, and Shiozaki, Nagano City). The samples were chosen according to availability and homogenized to obtain a clearer characterization of three varieties. By analyzing the samples of the same variety from different locations and cultivation environments, it was expected to understand the general characteristics of each type of sorghum.

The codification of each sample according to their place of cultivation is described in the table below (Table 1):

For the preparation of the samples for analysis, approximately 100 mg of bran were transferred to sample disruptor tubes supplied by Yasui Kikai (Osaka, Japan) and shaken with iron cones cooled in liquid nitrogen. Each sample was analyzed twice for the four platforms: CE/MS and LC/MS in both positive and negative polarities. The average of the two data sets was used as the expression value. Quality control (QC), containing all samples, was prepared, and analyzed every six measurements to ensure measurement accuracy. The coefficient of variance (CV%) was calculated for each metabolite using the QCs, and any metabolites with a CV% exceeding 50% were excluded from the data table.

The sample powders were then suspended in 1 mL of water and combined with 2 mL of methanol and 2 mL of chloroform. The mixture was shaken for 15 min and centrifuged at 1000× *g* for 10 min. The supernatants were carefully transferred to 15 mL conical centrifuge tubes and dried under a nitrogen stream at 40 °C. The dried residues were dissolved in 200 µL of a 10% acetonitrile and 90% water solution and subsequently analyzed using CE/MS and LC/MS.

#### 2.4.2. CE/MS Analysis and LC/MS Analysis

CE/MS experiments were conducted using an Agilent CE capillary electrophoresis system (Agilent Technologies, Waldbronn, Germany) coupled with an Agilent 6545 QTOF system (Agilent Technologies, Palo Alto, CA, USA). LC/MS analyses were conducted using an Agilent 1290 series UPLC system equipped with a 6545 quadrupole TOF system (Agilent Technologies, Palo Alto, CA, USA) controlled by MassHunter Workstation B.08.01 software. The analytical column employed was a CAPCELL PAK C18 IF, 2.0 mm ID × 50 mm, 2 µm (Osaka Soda, Osaka, Japan).

#### 2.4.3. CE/MS Analysis

For cationic separation, fused-silica capillaries (50 µm i.d., 100 cm total length) filled with 1 mol/L formic acid were used, while anionic separation utilized 20 mmol/L ammonium acetate and 20 mmol/L ammonium formate (pH 10) as the electrolyte. Sample solutions were injected at 50 mbar for 10 s, and a voltage of 30 kV was applied during electrophoresis. The capillary temperature was not controlled and maintained at room temperature, while the temperature of the sample tray was kept at 4 °C using an external thermostatic cooler. A sheath liquid of methanol and water (50% *v*/*v*) was delivered at a flow rate of 8 µL/min. ESI-TOF-MS analysis was performed in positive (cationic mode) and negative (anionic mode) ionization modes. The capillary voltage was set to 3 kV, and nitrogen gas (heater temperature 300 °C) was employed with a flow rate of 10 psi. Full scan exact mass data were acquired in the 60–1200 m/z range.

#### 2.4.4. LC/MS Analysis

The mobile phase consisted of eluent A, composed of water/ammonium acetate (5 mmol/L), and eluent B, composed of acetonitrile. Metabolites were eluted at a flow rate of 0.2 mL/min at 40 °C, utilizing a linear gradient from 10% to 100% of eluent B over 10 min, followed by a 5 min hold at 100% eluent B. The injection volume was set at 10 μL. Mass spectrometric analysis was performed in positive and negative ionization modes, with a scan rate of 2 spectra/s, covering a mass range of 60–1200 (m/z). The capillary voltage was set at 3500 V, and the fragment was adjusted to 120 V. The nebulizer operated at a pressure of 40 psi while the gas temperature was maintained at 350 °C. A continuous gas flow rate of 8 L/min was maintained throughout the analysis.

#### 2.4.5. LC/MS and CE/MS Data Processing

Data processing was conducted sequentially: (1) Peak picking: identifying all peak positions and areas. (2) Bias correction of retention time and m/z: correcting systematic errors or biases in retention time and m/z measurements using internal standards. (3) Peak alignment: aligning all peaks from different samples based on their m/z and retention time values. (4) Noise reduction: excluding noise level peaks in the data. (5) Bias correction of peak intensity: correcting systematic errors or biases in peak intensities using internal standards. (6) Peak identification: identifying metabolites using Marker analysis software (LSI Medience, Tokyo, Japan).

Isotopic, fragment, and adduct ions were eliminated from the peak datasets. The remaining peaks were compared across the samples and aligned based on m/z and retention time. Noise peaks in the samples were identified by comparison with blank preparation samples. The peaks detected in the samples were identified by matching their m/z values and normalized retention times with a set of 1379 standards obtained from reagent manufacturers.

The raw data obtained from the mass spectrometer were converted to CSV format using MassHunter Export software (version A.02.00) (Agilent Technologies, Santa Clara, CA, USA). The resulting CSV data included information on *m*/*z* (mass-to-charge ratio), retention time, and intensity. Principal component analysis (PCA) was performed using the extension XLSTAT 2019 from Microsoft Excel [33].

#### 2.4.6. Statistical Analysis

All analyses of colorimetric methods were conducted in triplicate, and the average and standard deviation were calculated. For the colorimetric methods, each variety had a sample collected from the same location. The package of each sample was then homogenized, and the extraction was performed in triplicate and each analysis was performed in triplicate. For the metabolomic analysis, as the goal was to access the main characteristics of each variety, each location had one homogenized sample.

The analysis of variance (ANOVA) was conducted using the extension XLSTAT 2019 (version 2019.2) from Microsoft Excel (Microsoft 365) [33] and *t*-test was also conducted on Microsoft Excel to evaluate the *p*-value and significance level among two different groups of samples.

## 3. Results

These results start with a brief explanation on the main phenotypic differences noticed among the samples chosen. It is followed by the quantification of the polyphenol content by colorimetric methods, and finally, the free flavonoids identified by untargeted metabolomics and their distinction according to the sorghum varieties and the growth environment.

### 3.1. Phenotypic Characteristics of the Studied Sorghum Varieties

The general characteristics of the crops are that TDN^®^ Sorgho can reach up to 2 m, Mini Sorgho can reach up to 1.5 to 1.8 m, and RILN-156 can reach 1.2 to 1.5 m. The sowing period is the same for all three varieties: April to August in warm areas, May to August in intermediate areas, and May to July in cold areas. Sorghum usually takes 60 to 80 days to reach its maximum height.

The three varieties differed in grain color, size, and shape. Thus, grains of the uncolored sorghum variety TDN^®^ were larger (length: 4.5 ± 0.28 mm, width: 4.0 ± 0.17 mm) than those of the colored ones (RILN-156 and MINI). The grains of the RILN-156 variety with the most intense color had the smallest sizes (3.5 ± 0.5 mm, 3.0 ± 0.7 mm). The dimensions (4.0 ± 0.5 mm, 3.5 ± 0.5 mm) and color of the MINI variety grains occupied an intermediate position. The size of the grains were positively proportional to the size of the crops.

**Figure 1:**
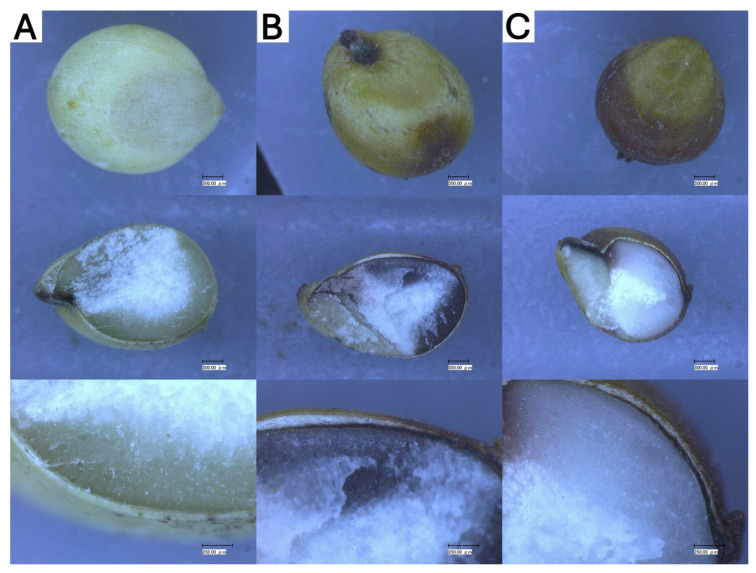
Microscopic observation of sorghum grains to represent the grains’ appearance as well as the presence of pigmented testa, thickness of pericarp, and secondary color. (**A**) TDN^®^ Sorgho, (**B**) Mini Sorgho, and (**C**) RILN-156. Scale 500.00 μm for the first and second lines and 500.00 μm for the third line (close-up images of the grains’ pigmented testa).

The varieties also present some distinctions regarding secondary color of the grains, presence of pigmented testa, and thickness of the pericarp (Figure 1). Contrary to colored sorghum, TDN^®^ Sorgho does not present a pigmented testa. As for both the red sorghums, although the floury endosperm—the white part in the middle of the grain—was observed to take different sizes in each grain from the same variety, the color of the corny endosperm (secondary color) of both types of grain is slightly different. Mini Sorgho presents a darker secondary color, closer to a brownish purple, while RILN-156 becomes similar to a whiteish red. By observing many samples of the grain, the thickness of the pericarp was very close between the red sorghum varieties and thinner in the white sorghum.

### 3.2. Determination of Polyphenolic Contents by Colorimetric Methods

This section will present the results of the investigation of free phenolic compounds soluble in methanol from the bran of the white sorghum sample (TDN^®^ Sorgho) and the two red sorghum samples (RILN-156 and Mini Sorgho). The reason why these chemical analyses were conducted, although a more accurate chromatographic analysis will be presented next, is that the samples were first analyzed over their general differences in the amount of main polyphenol contents and then analyzed by untargeted metabolomics, which due to the nature of the analysis could not be precisely quantified.

However, contrary to the untargeted metabolomics in which no pretreatment was performed on the samples, as the goal was to identify as many metabolites as possible, for the chemical analysis, a methanol extraction was conducted to better access the phenolic compound content of the samples. Therefore, the colorimetrical analyses were meant to have a general overview of the polyphenolic contents in the samples, while the untargeted metabolomics would be able to identify the specific components.

In this sense, despite both RILN-156 and Mini Sorgho being red sorghum varieties, the color of the polyphenolic extract from RILN-156 is more intense, closer to brown, in contrast to the orangish tone from the Mini extract.

We can observe in the following table (Table 2) of all the results obtained that Sorghum RILN-156 bran presented interesting values compared to the other two samples. RILN-156 had higher total phenolic content and flavonoid content. Corroborating with the untargeted metabolomics’ previously presented results, the total anthocyanin content, represented by apigenin and luteolin in sorghum, was the highest in white sorghum. Nevertheless, as expected, tannin was not detected in white sorghum but similar in Mini Sorgho and RILN-156, higher in Mini Sorgho bran. Except for tannin content, the bran of Mini Sorgho had the lowest concentrations. As for the scavenging assay with DPPH, RILN-156 presented the highest potential, followed by TDN^®^ Sorgho.

### 3.3. Analysis of the Data Obtained by Untargeted Metabolomic of Sorghum Bran

As this study aimed to perform an exploratory analysis to screen for potential biomarkers or compounds of interest, the focus was on identifying patterns of differential ion intensities between the samples of different varieties collected from different locations, rather than establishing absolute concentrations. As a result, it was possible to accomplish a broader discovery-based analysis, without the constraints of calibration-dependent quantification. The findings presented on this section, therefore, will serve as a foundation for future work, where targeted, quantitative methods, including the use of calibration curves will be employed to measure the concentrations of the specific analytes of interest that were able to be identified on this initial screening. Therefore, the Agilent 6545 Q-TOF was used since it stands out, especially in exploratory studies, due to its high-resolution accurate mass detection, MS/MS capabilities, and advanced software (MassHunter Export software, version A.02.00) integration.

This being said, by the untargeted metabolomics analysis using LC-MS and CE-MS, nineteen flavonoids were successfully identified (Table 3), where flavonols corresponded to 11% of the flavonoids identified among the three sorghum bran varieties.

Table 1 in the Section 2 contains the codification and the places of origin of each sample mentioned within the following data. From all 11 samples, malvidin was not present in RILN-156 (1), while epicatechin could not be detected in TDN^®^ Sorgho (1). Myricetin 3-rhamnoside was not identified in two out of the three TDN^®^ Sorgho samples: (1) and (2) and formononetin 7-glucoside was also non-detected in TDN^®^ Sorgho (1).

However, as one replicate was taken from each location, the main points are the flavonoids that were generally identified in each variety, being a common element in every location. Replicates from each location would require further investigation if these differences are due to the cultivation environment.

On the present identification, apigenin 8-glucoside and 7-glucoside were detected, but they were distinguished by the difference in LC elution time (about 0.3 min). The detected apigenin 7-glucoside was identified by comparing it with a standard specimen of O-glycoside.

In addition, a standard specimen of C-glycoside was used for apigenin 8-glucoside. Since we did not have a standard specimen of apigenin 7-C-glucoside, even if C-glucoside exists, we cannot state whether it can be distinguished from apigenin 7-O-glucoside. The same applies to apigenin 8-glucoside.

**Figure 2:**
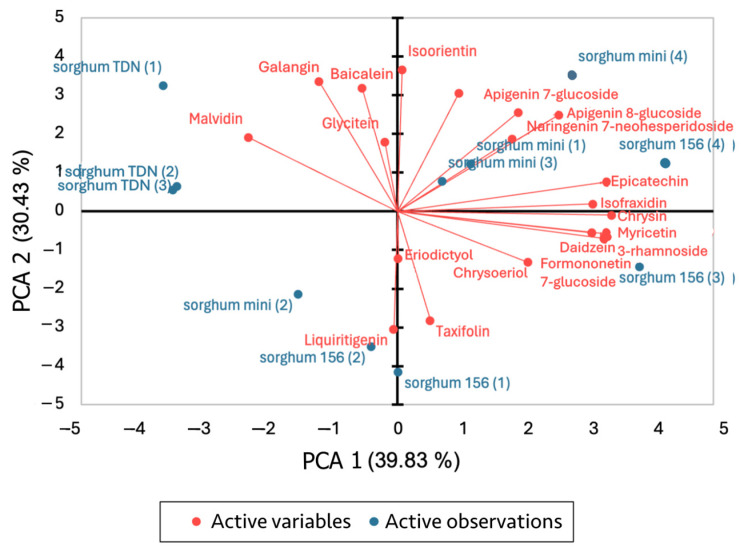
Graphic plots of PCA with the two axes totalizing 70.26% of the variances, where the plot on the left represents observations from the principal component analysis, and the plot from the right represents the biplot of active observations and variables. More data over the ratio of each component comparing varieties are expressed on Tables S1–S3 from the Supplementary Materials.

According to the different profiles regarding the concentration of flavonoids, principal component analysis (PCA) could be carried out using the differences of ion intensities, separating the sorghums, and determining the principal components among the flavonoids (Figure 2). By combining the two principal components, F1 and F2, this analysis could comprehend 70.26% of the total variance of the samples.

While conducting the PCA, the variables were determined as the flavonoid content identified. As, according to the location of collection of the samples, different concentrations and even absence of flavonoids were found, the variables amounted to 70%.

By looking at the PCA, we can observe that three of the four Mini Sorgho samples have similar flavonoid profiles, indicated by their high positive scores on both principal components. Mini Sorgho (2) and one of the sorghum samples, RILN-156 (2), are characterized by distinct flavonoid profiles compared to the other samples analyzed. In a general manner, contrasting with all the TDN^®^ Sorgho samples having similar flavonoid profiles with each other, the RILN-156 samples all presented variations among themselves, where RILN-156 (3) also has a distinct profile, RILN-156 (1) has an average profile that is not strongly influenced by either principal component, and RILN-156 (4) has a flavonoid profile characterized by the highest positive scores on both principal components (together with sorghum Mini Sorgho (4)).

As for the flavonoid influences in each sorghum, on the biplot with the variables, it can be observed that the TDN^®^ Sorgho samples were strongly associated with galangin, malvidin, and baicalein. Malvidin was the only anthocyanin that could be identified in this analysis. The flavonoid profile from sorghum mini (1), (3) and (4), and RILN-156 (4) is strongly influenced by apigenin-7-glucoside, naringenin-7-neohesperidoside, and apigenin-8-glucoside. Mini Sorgho (2) is associated with flavonoids like liquiritigenin, similar to RILN-156 (2), which eriodictyol also influences. As for the last two RILN-156 samples, RILN-156 (1) is associated with taxifolin but not strongly characterized by any other variables, while RILN-156 (3) is influenced by variables such as chrysin, formononetin-7-rhamnoside, and epicatechin.

**Figure 3:**
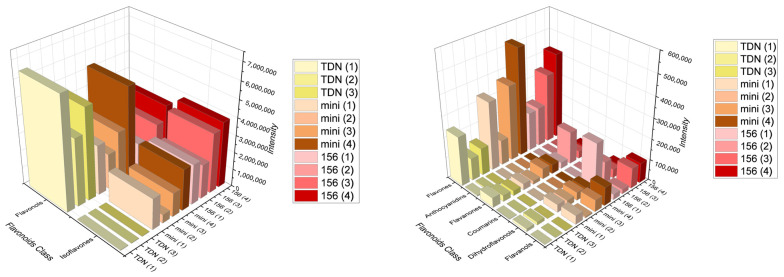
Distribution of the groups of flavonoids among the samples from different places according to the ion intensity observed. Left graph represents the most abundant classes identified while the right graph represents the minor classes.

**Figure 4:**
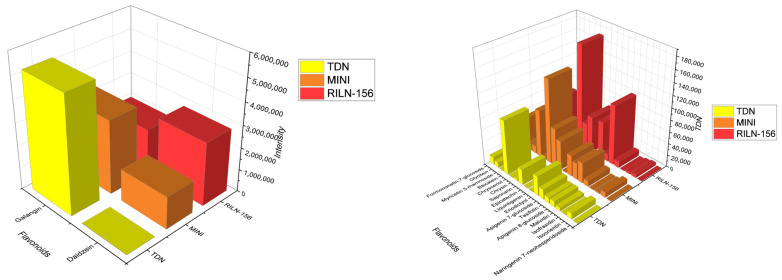
Distribution of each identified flavonoid among the samples from different places according to the ion intensity observed. Left graph represents the most abundant flavonoids identified while the right graph represents the minor flavonoids.

In this sense, we can observe that even if overall, the colored sorghums had a higher variety of polyphenolic compounds (Figure 3), the highest intensity was observed on white sorghum, with galangin (Figure 4).

**Figure 5:**
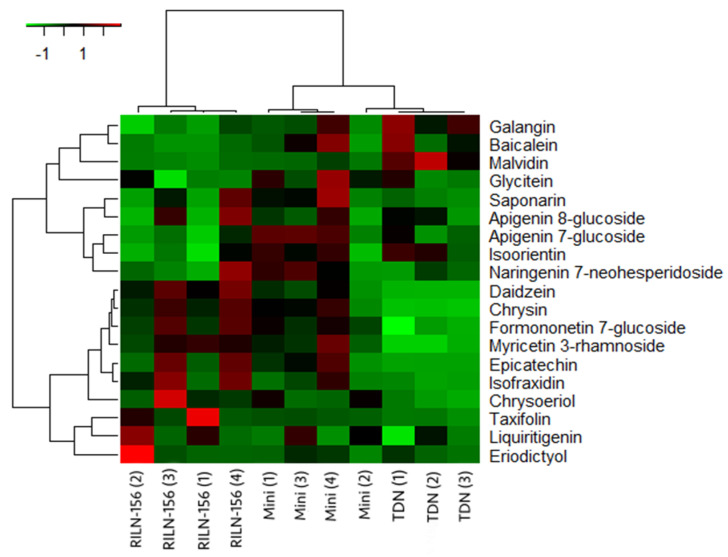
Heatmap of flavonoids identified by untargeted metabolomics analysis.

Furthermore, to better illustrate this, the heatmap below (Figure 5) shows that apart from the Mini Sorgho (2) sample, which presented a very different profile compared to the other sorghum samples, some groups of flavonoids are more abundant in red sorghum than on white sorghum. The clustering on the heatmap grouped the samples and flavonoids according to the similarity in their contents, so clear differences in “groups” of flavonoids can be observed. However, it is difficult to accurately affirm which factors could have affected the polyphenol content that had different concentrations within samples of the same variety that were collected from different places. These influencing factors could have arisen from the cultivation of the seed to the collection, treatment, and analysis of the sample. Additionally, environmental genetic factors could also play a role in these groups of flavonoids.

Correspondingly, except for flavonols and anthocyanin, red sorghum varieties presented a more significant intensity in flavonoids overall, indicating a higher concentration. Besides flavonols, Mini Sorgho and RILN-156 had a higher concentration of isoflavones and flavones as well. As mentioned, Taxifolin, representing the dihydroflavonols, was also outstanding in one of the RILN-156 samples. According to the concentrations observed by intensity values on untargeted metabolomics, galangin had the highest concentration among all the flavonoids. In the present work, galangin prevailed in white sorghum samples. The second most abundant flavonoid was daidzein. This isoflavone, however, was almost absent in TDN^®^ Sorgho bran, in contrast with the large amount present in the red sorghum samples.

Nonetheless, the bran of TDN^®^ Sorgho had a higher concentration of anthocyanin than the colored sorghum, even though it is a white variety. Despite the variations within the white sorghum samples collected from separate places, they were still higher in anthocyanin content than both colored varieties. On the other hand, Chrysin was higher in colored samples and barely present in the white sorghum bran. Baicalein, formononetin-7-glucoside, and eriodictyol also had significant concentrations.

## 4. Discussion

The concentration of phenolic compounds can be influenced by the grain’s genotype, which in turn influences the pericarp color and thickness, as well as the presence of colored testa in the grains and the secondary plant color (phenotype) [34]. In the present samples, we can observe that although both red pericarp sorghums present a pigmented testa and similar thickness, the secondary color of the grain is different.

Sorghums presenting red or purple as secondary colors have been reported to have higher phenolic concentrations in comparison with tan-colored sorghum [35]. By observing the appearance of the sorghum grains cut in half, we could observe that Mini Sorgho has a brownish purple secondary color, while RILN-156 has a whiteish red. White sorghum has a tan secondary color.

On the investigation of the general quantification of polyphenolic compounds using colorimetric methods, RILN-156 bran presented the highest value of phenolic content (91.8 μg.GAE/g of bran), while Mini Sorgho presented 42.2 μg.GAE/g. Usually, white sorghum presents a lower concentration of phenolic compounds than colored sorghum [14], contrary to what happened in this work (TDN^®^ Sorgho presented 55.6 μg.GAE/g). We can observe that both red sorghums presented a significant difference in the extraction of polyphenol content and flavonoids using methanol, 70%. In contrast, Mini Sorgho presented results that were lower than those of TDN^®^ Sorgho. This could be due to the presence of a large concentration of flavonoids and polyphenols that do not have as much affinity with the solvent as the ones present in RILN-156 since the solvent polarity is important in the determination of which components will be extracted [36].

Likewise, in the determination of total flavonoid contents, RILN-156 once again had the highest concentration (1196.3 μg.QE/g), followed by TDN^®^ Sorgho (413.2 μg.QE/g) and Mini Sorgho (185.1 μg.QE/g). The results obtained by RILN-156 corroborate previous studies that reported that colored sorghums present higher levels of secondary metabolites such as flavonoids [37].

As for anthocyanin content, luteolin and apigenin were taken as references. Surprisingly, among the free phenolic compounds quantified, TDN^®^ Sorgho was the sample with the highest concentration of both luteolin (6231.9 μg/g) and apigenin (4290.5 μg/g). RILN-156 had a significantly lower concentration (486.7 μg/g and 335.3 μg/g), as well as Mini Sorgho (151.8 μg/g and 104.6 μg/g).

While methanol is often employed for the extraction of anthocyanin and the addition of water could improve its yield due to similar polarity, other conditions, such as temperature and time, should be optimized for focusing on anthocyanin extraction [38], besides the possible interaction with flavones, which could also be one hypothesis to explain the differences in these results for red sorghum, which will be further discussed with the untargeted metabolomics results.

Total condensed tannin was not detected in white sorghum but presented slight variation among the colored sorghum samples. Mini Sorgho bran (1722 μg.CE/g), followed by RILN-156 (1149 μg.CE/g), showed a substantial content of tannins. Despite acting as antioxidants, tannins are also considered anti-nutrients due to their possible inhibition of proteins and their influence on the digestibility of some amino acids [39].

Although high tannin content was observed in the colorimetric analysis, no tannin was identified during LC/MS or CE/MS analysis. This can be due to a few reasons, such as the previous methanol extraction performed on the samples analyzed by colorimetric methods in contrast to the natural samples used in the LC/MS and CE/MS analyses. During LC/MS and CE/MS, tannin could have bound to other matrix components causing interferences in its detection. Additionally, matrix effects could have suppressed the ionization of tannin and prevented its detection. However, tannin precursors such as epicatechin were identified within red sorghum samples by LC/MS and CE/MS, which could indicate the presence of tannin as well.

Sorghums that contain tannin are known to be resistant to birds and insects and provide a higher yield, which might influence farmers’ choices depending on the application it will be destined for [40]. For dietary purposes, it is a positive result that the newly developed sorghum obtained a lower tannin content. More tests in vitro would be necessary to imply that the amount of tannin present could contribute to the bioactivity of the grain while not being as abundant as to have a heavy influence on the absorption of the compounds of interest.

On DPPH scavenging activity, the colored sorghum samples, Mini Sorgho and RILN-156, had 106.4 mM.TE/g and 266.4 mM.TE/g, respectively, while the white sorghum TDN^®^ Sorgho had 230 mM.TE/g as scavenging activity. A higher phenolic content means higher antioxidant activity since phenolics and flavonoid molecules have been shown to have a high correlation with the antioxidant activity of plant extracts [41]. Based on this, the DPPH scavenging activity values obtained from the current samples were as high as the total phenolic contents were.

For the human body, a component with better antioxidant activity in a food product means a higher chance of reducing the risk of degenerative diseases, such as cancer. Naturally, many factors affect such diseases. However, studies have proven the mechanism of action of antioxidant compounds in heart and respiratory diseases, arthritis, inflammatory diseases, and even Parkinson’s disease [42]. In this sense, in vitro and in vivo tests with bioactive antioxidant components extracted from sorghum bran have been conducted for more than a decade now [43,44], indicating that sorghum bran, especially colored varieties, could be a great addition to the human diet depending on the bioavailability of such components.

As mentioned in the Section 3, under the untargeted metabolomics analysis, flavonols were the larger class of flavonoids identified within the three sorghum bran varieties. Flavonols, one of the most abundant classes of flavonoids, represented by quercetin, have been gaining attention in the food and pharmaceutical industries, especially now that the search for natural healthy compounds and functional foods is standing out. Structurally similar to flavones, flavonols are colorless compounds with an extra non-phenolic hydroxyl group at position 3 [45]. They have a wide range of roles in the plant defense system, including being responsible for the plant–microbe interaction and protection from UV rays and microbial attacks [46].

Galangin had the highest concentration among all the flavonoids, especially on TDN^®^ Sorgho. Galangin, a 3,5,7-trihydroxyflavone, is usually found in honey and propolis and has been investigated to prevent diverse diseases and human conditions like aging and inflammation [47].

Despite being abundant in the samples, galangin has been reported not to be toxic up to 5 g/kg and not to cause any side effects [48]. After ingestion, galangin can be metabolized into kaempferol and quercetin, two important antioxidants [49]. Such traits enable the study of its antiproliferative ability against several types of cancer cells, like esophageal [50], leukemia [51], and even skin cancer [52].

The second most abundant flavonoid was daidzein. This isoflavone, however, was almost absent in TDN^®^ Sorgho bran in contrast with large amounts present in the red sorghum samples. Primarily found in soybeans, daidzein has many roles in the biotic and abiotic stress defense mechanism of plants, such as influencing the receptivity of symbiotic root infection, defense against oxidative stress, and so on [53].

Being chemically similar to mammalian estrogens, daidzein presents estrogenic properties that can be beneficial by hindering or substituting estrogen and estrogen receptor complex, protecting against diseases related to the control of estrogens, like breast cancer, diabetes, osteoporosis, and cardiovascular disease [54]. In a study with soybeans, it was noticed that the daidzein content increased under waterlogging [55], which also shows that although daidzein seems to be a flavonoid abundant in colored sorghums, it can also be influenced by conditions such as water stress.

In contrast, the concentration of anthocyanin, represented by the anthocyanidin malvidin, was higher on TDN^®^ Sorgho bran than the colored sorghum bran despite being a white variety. Although, to our knowledge, it has not yet been reported in sorghum, it is not uncommon. Many factors can modify the color of anthocyanins, from genetic traits to environmental conditions. Apart from the genetic, environmental, and sample manipulation factors from cultivation until analysis that could have affected the exact amount of malvidin produced, they still had higher anthocyanin content than both colored varieties.

One hypothesis would be sorghum’s high production of flavones, considered co-pigments. In some plants, flavones and anthocyanins interact in inversely proportional ways: the more flavones produced, the fewer anthocyanins are present. Additionally, anthocyanins’ color or intensity will change depending on the amount of hydrogen ions in flavones [56]. However, we noticed that, in the present work, even when some of the samples of colored varieties presented a similar concentration of flavones as the white varieties, the anthocyanidin content did not increase.

**Figure 6:**
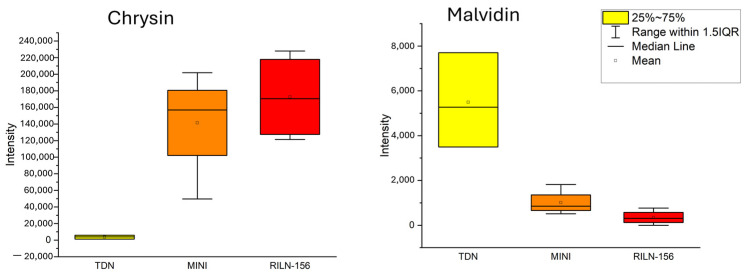
Boxplot graph comparing the concentration of malvidin and chrysin in the samples. Colors are illustrative: yellow represent the white sorghum, while orange and red represent the red sorghum varieties.

Nonetheless, we cannot rule out that a specific flavone could interact with malvidin. For example, white sorghum bran samples had a smaller chrysin content than their anthocyanidin concentration. In contrast, red sorghum brans presented a significant amount of the same flavone (Figure 6). Although further investigation would be necessary to confirm the interaction between these two metabolites, it is still interesting to notice this coincidental pattern.

Chrysin (5,7-dihydroxyflavone), besides the common properties of flavonoids, has also been studied regarding its antispasmodic and anxiolytic properties [57]. On the other hand, the anthocyanidin malvidin is extensively known for helping in the attribution of the color of red grapes and wine [58]. As malvidin has been thoroughly investigated, it has been reported to have anticarcinogenic, diabetes control, cardiovascular-disease-prevention, and brain-function-improvement properties [59].

Baicalein, which was also slightly significant in the samples, comes from the chrysin biosynthetic pathway and has been investigated for its contribution to preventing cancer and diseases [60]. Baicalein has also recently been investigated for treating SARS-CoV-2 [61]. In addition, chrysoeriol, taxifolin, and eriodictyol were most present in RILN-156. That is a promising result for this newly developed inbred line.

Although in smaller concentrations, the other identified flavonoids have also been reported to contribute to health in in vitro and in vivo tests.

As previously reported in other works [62], we could observe that despite RILN-156 having a more significant amount of polyphenol, other factors considerably affect the polyphenolic profile of the grain. Nevertheless, it is essential to conduct bioavailability tests to ensure that those components can be metabolized by the human body during digestion, as around 80% of sorghum bioactive components are covalently bound to other cell wall compounds, like arabinoxylan [63].

The promising results of RILN-156 show feasibility of conducting further in vitro tests with the new variety. Although when compared in Table S3, both colored sorghums were not statistically different, each of them can be chosen according to their availability. White sorghum could also be used for applications that do not require tannin content.

As the present work obtained its data of general quantification of phenolic compounds after a methanol extraction, it is necessary to analyze extractions performed on a more similar environment to the human body, to understand more of its bioavailability. Similar compounds as the flavonoids found on the present work were found on supercritical water extraction and ultrasound extraction from sorghum bran, giving an insight about its bioavailability [64,65]. Tannins from sorghum have been reported to reduce glycemic response, after being ingested in a sorghum-based drink [66]. Sorghum bran extracted with supercritical water was also analyzed on its ability to inhibit cells from liver cancer (HepG2 cells) and an increase in the inhibition was noticed [65].

In other cases, tannins from sorghum (30 mg/g) significantly affected the absorption of other nutrients during in vitro tests and fermentation by microorganisms, when simulations on a porcine gastrointestinal tract were carried out [67]. As the samples from the present work present lower concentrations than 30 mg/g, it might imply that different inhibition proportions could be found in in vitro tests.

Regarding the chemical structure, flavanones and flavones, that were identified in the present work, usually have a high bioavailability due to their O-glucosides form, that make them highly hydrolysable [68]. Daidzein, also known as genistein, which has presented a high concentration on the red sorghum samples analyzed, despite having been reported to have low oral availability, has many clinical trials aiming to improve its availability. Perhaps red sorghum samples could also become a subject of study on daidzein chemical disposition in sorghum composition and consequently bioavailability [69].

In general, studies have shown that in vitro gastrointestinal digestion in processed sorghum affected the bioaccessibility of phenolic compounds and that the grain’s growing location impacted the antioxidant activity and phenolic content of the samples studied, even in compounds that were easily accessible. Moreover, processed sorghums successfully reach the colon in the gastrointestinal tract, promoting health benefits [70].

The current data briefly accessed the general flavonoid profile and potential of the newly developed variety among two commercially available varieties in Nagano Prefecture, Japan. In addition, a short insight on the distinctions on flavonoid profile within varieties could be accessed. It is important to consider that even though the polyphenol content might be greatly affected by environmental characteristics, the state of ripeness during harvesting, processing, and storage, as well as soil type, rainfall, type of culture, and exposure to light will also affect the production of these components in the plant. What is more, exposure to light is a crucial affecting factor in flavonoids [71].

Additionally, although sorghum has great potential for many applications, there are still some challenges for its use in the industry, which reaffirms the need for studies of different varieties and their composition [68].

## 5. Conclusions

This initial screening provided insight on the flavonoid profile of the bran of the sorghum varieties investigated. The recombinant inbred line RILN-156 had a greater antioxidant scavenging potential than the two other varieties analyzed, which showed favorable potential for using this inbred line from now on. Common characteristics among colored and non-colored samples were also observed, such as the predominance of galangin in TDN^®^ Sorgho bran and daidzein in the colored samples.

Overall, when compared with the two commercially available sorghum brans, having a rich flavonoid profile and antioxidant activity disclosed by the present study, the sorghum bran of the newly developed variety has the potential for future utilization industrially and even commercialization. Further targeted analysis and quantification analysis by chromatographic methods will be carried out now that the exploratory analysis has been completed.

## Figures and Tables

**Table 1:** Sorghum codification by place of origin.

TDN^®^ Sorgho	Iizuna Town	TDN (1)
	Naniai—Nagano City	TDN (2)
	Wakaho—Nagano City	TDN (3)
Mini Sorgho	Chikuma City	mini (1)
	Nagano City	mini (2)
	Shiojiri City	mini (3)
	Suzaka City	mini (4)
RILN-156	Naniai—Nagano	156 (1)
	Shiozaki, Nagano City	156 (2)
	Suzaka City	156 (3)
	Ueda City	156 (4)

**Table 2:** Results obtained from the methanol extraction of TDN^®^, Mini, and RILN-156 bran.

Analysis		TDN^®^ Sorgho	Mini Sorgho	RILN-156	*p*-Value		
					TDN vs. MINI	TDN vs. 156	Mini vs. 156
Total phenolic content (μg.GAE/g)		55.6 ± 0.008	42.2 ± 0.001	91.8 ± 0.007	9.10 × 10^−8^	2.54 × 10^−10^	4.88 × 10^−9^
Total flavonoid content (μg.QE/g)		413.2 ± 0.010	185.1 ± 0.001	1196.3 ± 0.071	5.19 × 10^−10^	1.74 × 10^−6^	1.05 × 10^−6^
Total anthocyanin content	Apigenin (μg/g)	4290.5 ± 0.008	104.6 ± 0.004	335.3 ± 0.014	3.74 × 10^−5^	4.12 × 10^−5^	2.76 × 10^−5^
	Luteolin (μg/g)	6231.9 ± 0.002	151.8 ± 0.005	486.7 ± 0.006	8.12 × 10−^−14^	1.62 × 10^−13^	2.97 × 10^−12^
Total tannin content (μg.CE/g)		N.D.	1722 ± 0.139	1149 ± 0.155	*	*	2.60 × 10^−10^
DPPH scavenging assay (mM.TE/g)		230 ± 6.313	106.4 ± 5.214	266.4 ±1.714	5.66 × 10^−3^	8.00 × 10^−2^	1.39 × 10^−2^

ND: non-detected; * Could not be compared since analysis conducted on TDN^®^ Sorgho did not appoint the presence of tannin; the values are expressed in mean values ± standard deviation. After conducting ANOVA and *t*-test, at the 0.05 level, the population means are significantly different for all analysis.

**Table 3:** Flavonoids identified by LC/MS and CE/MS.

RT (min)	Mass	No. of Samples	Samples	Compound Name	Fragmentation	Observed aΔ [ppm]	Flavonoid Class	Molecular Formula	Method of ID
8.7	579.176	11	TDN 1, 2, 3; MINI 1, 2, 3; RILN-156 1, 2, 3.	Naringenin 7-neohesperidoside	[M-H]−	−5.2	Flavanone	C_27_H_32_O_14_	Metabolite CE AN
4.53	447.092	11	TDN 1, 2, 3; MINI 1, 2, 3; RILN-156 1, 2, 3.	Isoorientin	[M-H]−	0.0	Flavone	C_21_H_20_O_11_	Metabolite LC Neg
4.9	221.050	11	TDN 1, 2, 3; MINI 1, 2, 3; RILN-156 1, 2, 3.	Isofraxidin	[M-H]−	22.6	Coumarin	C_11_H_10_O_5_	Metabolite LC Neg
4.97	347.105	10	TDN 1, 3; MINI 1, 2, 3; RILN-156 1, 2, 3.	Malvidin	[M-H+NH3]−	2.9	Anthocyanidin	C_17_H_15_O_7_+	Metabolite LC Neg
5.01	431.095	11	TDN 1, 2, 3; MINI 1, 2, 3; RILN-156 1, 2, 3.	Apigenin 8-glucoside	[M-H]−	−4.6	Flavone	C_27_H_30_O_14_	Metabolite LC Neg
5.05	303.047	11	TDN 1, 2, 3; MINI 1, 2, 3; RILN-156 1, 2, 3.	Taxifolin	[M-H]−	−9.9	Dihydroflavonol	C_15_H_12_O_7_	Metabolite LC Neg
5.28	431.091	11	TDN 1, 2, 3; MINI 1, 2, 3; RILN-156 1, 2, 3.	Apigenin 7-glucoside	[M-H]−	−16.2	Flavone	C_21_H_20_O_10_	Metabolite LC Neg
5.97	283.060	11	TDN 1, 2, 3; MINI 1, 2, 3; RILN-156 1, 2, 3.	Glycitein	[M-H]−	−3.5	Isoflavone	C_16_H_12_O_5_	Metabolite LC Neg
6.15	287.051	11	TDN 1, 2, 3; MINI 1, 2, 3; RILN-156 1, 2, 3.	Eriodictyol	[M-H]−	−13.9	Flavanone	C_15_H_12_O_6_	Metabolite LC Neg
6.24	255.068	11	TDN 1, 2, 3; MINI 1, 2, 3; RILN-156 1, 2, 3.	Liquiritigenin	[M-H]−	11.8	Flavanone	C_15_H_12_O_4_	Metabolite LC Neg
8.24	269.044	11	TDN 1, 2, 3; MINI 1, 2, 3; RILN-156 1, 2, 3.	Galangin	[M-H]−	−3.7	Flavonol	C_15_H_10_O_5_	Metabolite LC Neg
3.93	291.084	10	TDN 2, 3; MINI 1, 2, 3; RILN-156 1, 2, 3.	Epicatechin	[M+H]+	−10.3	Flavanol	C_15_H_14_O_6_	Metabolite LC Pos
4.3	595.173	11	TDN 1, 2, 3; MINI 1, 2, 3; RILN-156 1, 2, 3.	Saponarin	[M+H]+	11.8	Flavone	C_27_H_30_O_15_	Metabolite LC Pos
4.99	465.105	9	TDN 3; MINI 1, 2, 3; RILN-156 1, 2, 3.	Myricetin 3-rhamnoside	[M+H]+	4.3	Flavonol	C_21_H_20_O_12_	Metabolite LC Pos
5.83	431.130	10	TDN 1, 2, 3; MINI 1, 2, 3; RILN-156 1, 2, 3.	Formononetin 7-glucoside	[M+H]+	−9.3	Isoflavone	C_22_H_22_O_9_	Metabolite LC Pos
5.94	255.061	11	TDN 1, 2, 3; MINI 1, 2, 3; RILN-156 1, 2, 3.	Daidzein	[M+H]+	−19.6	Isoflavone	C_15_H_10_O_4_	Metabolite LC Pos
6.83	255.061	11	TDN 1, 2, 3; MINI 1, 2, 3; RILN-156 1, 2, 3.	Chrysin	[M+H]+	−11.8	Flavone	C_15_H_10_O_4_	Metabolite LC Pos
6.88	301.071	11	TDN 1, 2, 3; MINI 1, 2, 3; RILN-156 1, 2, 3.	Chrysoeriol	[M+H]+	0.0	Flavone	C_16_H_12_O_6_	Metabolite LC Pos
7.01	271.057	11	TDN 2, 3; MINI 1, 2, 3; RILN-156 1, 2, 3.	Baicalein	[M+H]+	−11.1	Flavone	C_15_H_10_O_5_	Metabolite LC Pos

UV measurement was not performed. The tentatively predicted compounds were the same as the identified compounds. RT: retention time; CE: capillary electrophoresis; LC: liquid chromatography; AN: anion; Neg: negative; Pos: positive.

## Data Availability

The original contributions presented in the study are included in the article/Supplementary Materials, further inquiries can be directed to the corresponding author.

## Supplementary Materials

The following supporting information can be downloaded at https://www.mdpi.com/article/10.3390/metabo14110628/s1: Table S1. Comparison among TDN Sorgho samples and Mini Sorgho; Table S2. Comparison among TDN Sorgho samples and RILN-156; Table S3. Comparison among Mini Sorgho samples and RILN-156.
