# Supplementary material for: Comparison of Free Flavonoids and the Polyphenol Content in the Bran of a Newly Developed Sorghum Variety and Two Commercially Available Sorghum Varieties
Source: Metabolites. 2024 Nov 15;14(11):628. doi: 10.3390/metabo14110628 (PMC11596866; doi:10.3390/metabo14110628)
Supplement: Supplementary file 1 [file metabolites-14-00628-s001.zip › metabolites-3273576-supplementary.pdf]

Supplementary Materials

Article

Comparison of Free Flavonoids and the Polyphenol Content in the Bran of a Newly Developed Sorghum Variety and Two Commercially Available Sorghum Varieties

Table S1. Comparison among TDN Sorgho samples and Mini Sorgho. Mean (light blue): Mean value of control; SD (light blue): Standard deviation of control; Mean (yellow): Mean value of experimental group; SD (yellow): Standard deviation of experimental group; P Value: T-test P value; T-TEST: *: < 0.05, **: < 0.01; Separation: *: Cases where the two groups are completely separated; Ratio: Ratio of variation of experimental group against control (P<0.05: color display).

Name	Mean	SD	Mean	SD	P Value	T-TEST	Separation	Ratio (tdn/mini)
Naringenin 7-neohesperidoside	167	82	337	178	1.9E-01			2.01
Isoorientin	9606	2862	8775	3993	7.7E-01			0.91
Isofraxidin	1146	159	2033	824	1.3E-01		*	1.77
Malvidin	5490	2114	1009	566	8.7E-03	**	*	0.18
Apigenin 8-glucoside	9885	3846	9551	4369	9.2E-01			0.97
Taxifolin	9366	7934	34315	4961	3.6E-03	**	*	3.66
Apigenin 7-glucoside	16898	7683	27703	10635	2.0E-01			1.64
Glycitein	8750	3171	11993	3265	2.5E-01			1.37
Eriodictyol	33085	12039	35366	15385	8.4E-01			1.07
Liquiritigenin	329	154	432	138	3.9E-01			1.31
Galangin	5259767	1314712	3326257	1409575	1.2E-01			0.63
Epicatechin	346	303	48968	34476	6.7E-02		*	141.66
Saponarin	26114	6765	64487	36595	1.4E-01			2.47
Myricetin 3-rhamnoside	733	1269	12687	5739	1.8E-02	*	*	17.32
Formononetin 7-glucoside	13044	11499	51511	9451	4.6E-03	**	*	3.95
Daidzein	7970	11359	1455403	792133	3.5E-02	*	*	182.60
Chrysin	3662	2409	141285	64749	2.4E-02	*	*	38.58
Chrysoeriol	1014	738	3993	1955	5.7E-02		*	3.94
Baicalein	91712	53900	80031	54652	7.9E-01			0.87

Table S2. Comparison among TDN Sorgho samples and RILN-156. Mean (light blue): Mean value of control; SD (light blue): Standard deviation of control; Mean (yellow): Mean value of experimental group; SD (yellow): Standard deviation of experimental group; P Value: T-test P value; T-TEST: *: < 0.05, **: < 0.01; Separation: *: Cases where the two groups are completely separated; Ratio: Ratio of variation of experimental group against control (P<0.05: color display).

Name	Mean	SD	Mean	SD	P Value	T-TEST	Separation	Ratio (tdn/156)
Naringenin 7-neohesperidoside	167	82	236	248	6.7E-01			1.41
Isoorientin	9606	2862	4993	3298	1.1E-01			0.52
Isofraxidin	1146	159	2988	1198	5.3E-02		*	2.61
Malvidin	5490	2114	348	322	5.0E-02	*	*	0.06
Apigenin 8-glucoside	9885	3846	10539	7589	9.0E-01			1.07
Taxifolin	9366	7934	101007	87832	1.3E-01		*	10.78
Apigenin 7-glucoside	16898	7683	12138	6775	4.2E-01			0.72
Glycitein	8750	3171	7208	2977	5.4E-01			0.82
Eriodictyol	33085	12039	62550	63076	4.7E-01			1.89
Liquiritigenin	329	154	499	173	2.4E-01			1.52
Galangin	5259767	1314712	2046769	919369	1.2E-02	*	*	0.39
Epicatechin	346	303	60830	42680	6.6E-02		*	175.98
Saponarin	26114	6765	44651	38274	4.5E-01			1.71
Myricetin 3-rhamnoside	733	1269	14655	3741	1.8E-03	**	*	20.00
Formononetin 7-glucoside	13044	11499	58991	18215	1.3E-02	*	*	4.52
Daidzein	7970	11359	2837786	865983	7.3E-03	**	*	356.04
Chrysin	3662	2409	172591	53097	7.8E-03	**	*	47.13
Chrysoeriol	1014	738	5494	4048	1.2E-01		*	5.42
Baicalein	91712	53900	32980	9063	2.0E-01			0.36

Table S3. Comparison among Mini Sorgho samples and RILN-156. Mean (light blue): Mean value of control; SD (light blue): Standard deviation of control; Mean (yellow): Mean value of experimental group; SD (yellow): Standard deviation of experimental group; P Value: T-test P value; T-TEST: *: < 0.05, **: < 0.01; Separation: *: Cases where the two groups are completely separated; Ratio: Ratio of variation of experimental group against control (P<0.05: color display).

Name	Mean	SD	Mean	SD	P Value	T-TEST	Separation	Ratio (mini/156)
Naringenin 7-neohesperidoside	337	178	147	248	5.4E-01			2.30
Isoorientin	8775	3993	4414	3298	2.0E-01			1.99
Isofraxidin	2033	824	3120	1198	2.4E-01			0.65
Malvidin	1009	566	311	322	1.0E-01			3.25
Apigenin 8-glucoside	9551	4369	9661	7589	8.3E-01			0.99
Taxifolin	34315	4961	74163	87832	2.3E-01			0.46
Apigenin 7-glucoside	27703	10635	11534	6775	5.6E-02			2.40
Glycitein	11993	3265	6934	2977	7.4E-02			1.73
Eriodictyol	35366	15385	32569	63076	4.6E-01			1.09
Liquiritigenin	432	138	466	173	5.7E-01			0.93
Galangin	3326257	1409575	2023244	919369	1.9E-01			1.64
Epicatechin	48968	34476	61485	42680	6.8E-01			0.80
Saponarin	64487	36595	35538	38274	4.8E-01			1.81
Myricetin 3-rhamnoside	12687	5739	16096	3741	5.9E-01			0.79
Formononetin 7-glucoside	51511	9451	59269	18215	5.0E-01			0.87
Daidzein	1455403	792133	2842888	865983	5.7E-02			0.51
Chrysin	141285	64749	170540	53097	4.8E-01			0.83
Chrysoeriol	3993	1955	3934	4048	5.4E-01			1.01
Baicalein	80031	54652	30969	9063	1.8E-01			2.58
